# Activation of SphK1 by adipocytes mediates epithelial ovarian cancer cell proliferation

**DOI:** 10.1186/s13048-021-00815-y

**Published:** 2021-05-01

**Authors:** Lan Dai, Chen Wang, Keqi Song, Wenjing Wang, Wen Di

**Affiliations:** 1 Department of Obstetrics and Gynecology, Ren Ji Hospital, School of Medicine, Shanghai Jiao Tong University, Shanghai, 200127 China; 2 Shanghai Key Laboratory of Gynecologic Oncology, Ren Ji Hospital, School of Medicine, Shanghai Jiao Tong University, Shanghai, 200127 China; 3 State Key Laboratory of Oncogene and Related Genes, Shanghai Cancer Institute, Ren Ji Hospital, School of Medicine, Shanghai Jiao Tong University, Shanghai, 200127 China

**Keywords:** Epithelial ovarian cancer (EOC), Adipocytes, Sphingosine kinase 1 (SphK1), Sphingosine 1-phosphate receptor (S1PR), Proliferation

## Abstract

**Background:**

Adipocytes, active facilitators of epithelial ovarian cancer (EOC) growth, have been implicated in the link between obesity and EOC. However, the current understanding of the mechanisms underlying adipocyte-induced EOC cell proliferation remains incomplete.

**Results:**

We provide the first evidence showing that sphingosine kinase (SphK) 1 is critical for adipocyte-induced EOC cell proliferation. Adipocytes are capable of activating SphK1, which then leads to extracellular signal-regulated kinase (ERK) phosphorylation. Moreover, adipocyte-induced SphK1 activation is ERK dependent. Furthermore, sphingosine 1-phosphate receptor (S1PR) 1 and S1PR3, key components of the SphK1 signalling pathway, participate in adipocyte-mediated growth-promoting action in EOC cells.

**Conclusions:**

Our study reveals a previously unrecognized role of SphK1 in adipocyte-induced growth-promoting action in EOC, suggesting a new target for EOC therapy.

## Background

Obesity is a growing health concern worldwide, and there is increasing evidence for an association between obesity and epithelial ovarian cancer (EOC). For instance, epidemiological evidence indicates that the risk of EOC increases with increasing body mass index (BMI) [1]. The risk of EOC is higher among overweight women and even higher among obese women than among women with a “healthy” BMI [2]. Moreover, the survival of obese women with ovarian cancer is worse than that of nonobese women [2]. Although the mechanisms underlying the link between obesity and EOC remain unclear, it is certain that adipocytes, key cells during the development of obesity, play critical roles in the growth of EOC [3, 4]. Previous studies have shown that adipocytes can promote the growth of EOC by transferring nutrients to EOC cells and activating lipid metabolism in these cells [3]. However, the mechanisms critical for the growth-promoting effect of adipocytes in EOC remain to be discovered.

Sphingosine kinase (SphK) 1, an enzyme that catalyses sphingosine phosphorylation, is a critical mediator of numerous signalling networks and plays key roles in tumour progression [5–7]. SphK1 can be activated by a range of stimuli, such as growth factors and inflammatory cytokines [5–7]. Its activation was reported to contribute to the survival, proliferation, migration, invasion, and drug resistance of various cancer types [5–7]. We previously showed that SphK1, which is overexpressed in EOC tissue [8], is required for EOC metastasis and angiogenesis [8, 9] and that its activation is inversely correlated with survival in EOC patients [10]. Although SphK1 is a significant signalling enzyme in EOC progression, its regulatory mechanisms are unclear. In this work, we provide the first evidence showing that adipocytes are able to activate SphK1 and reveal a previously unrecognized role of SphK1 in the adipocyte-induced growth-promoting action in EOC.

## Materials and methods

### Reagents and antibodies

Antibodies against ERK1/2 and phospho-ERK1 (Thr202/Tyr204)/ERK2 (Thr185/Tyr187) were purchased from Cell Signaling Technology. Antibodies against phospho-SphK1 (Ser225) were purchased from ECM Biosciences. Antibodies against SphK1, S1PR1, S1PR2 and S1PR3 were purchased from Abcam. GAPDH antibody was ordered from Sigma-Aldrich. U0126, PF543, insulin, dexamethasone and 3-isobutyl-1-methylxanthine were ordered from Sigma-Aldrich.

### Cell lines and cell culture

The SKOV3 human EOC cell line was purchased from American Type Culture Collection and cultured in DMEM (Invitrogen) supplemented with 10% foetal bovine serum (FBS). The A2780 human EOC cell line was obtained from the China Center for Type Culture Collection and cultured in DMEM supplemented with 10% FBS. The murine 3T3-L1 preadipocyte cell line was obtained from the Cell Bank of Chinese Academy of Sciences and cultured in DMEM supplemented with 10% calf serum. 3T3-L1 preadipocytes were induced into mature adipocytes by treatment with insulin, dexamethasone and 3-isobutyl-1-methylxanthine as described previously [11]. To generate adipocyte-conditioned medium (Adi-CM), mature adipocytes were cultured with serum-free medium (SFM) for 24 h. The Adi-CM was then collected and filtered.

### Small interfering RNAs (siRNAs) and transient transfection

Chemically synthesized siRNAs targeting human SphK1 (5′-AAGAGCUGCAAGGCCUUGCCC-3′), S1PR1 (5′-AAGCUACACAAAAAGCCUGGA-3′), S1PR2 (5′-AAUACCUUGCUCUCUGGCUCU-3′), S1PR3 (5′-CUGCCUGCACAAUCUCCCUTT-3′) and the control siRNA (5′-AAUUCUCCGAACGUGUCACGU-3′) were ordered from GenePharma [9]. Lipofectamine (Invitrogen) was used for siRNA transfection. The levels of targeted genes were tested by RT-PCR and western blot analysis after transfection.

### Real-time RT-PCR

RNA was extracted using TRIzol Reagent (Invitrogen). The mRNA levels were measured with SYBR Green RT-PCR and then calculated by the 2^-ΔΔCt^ method. The following primers were used: SphK1, 5′-CATTATGCTGGCTATGAGCAG-3′ (forward) and 5′-GTCCACATCAGCAATGAAGC-3′ (reverse); S1PR1, 5′-CCTCTTCCTGCTAATCAGCG-3′ (forward) and 5′-ACAGGTCTTCACCTTGCAGC-3′ (reverse); S1PR2, 5′-CATTGCCAAGGTCAAGCTGT-3′ (forward) and 5′-ACGATGGTGACCGTCTTGAG-3′ (reverse); S1PR3, 5′-TCAGCCTGTCTCCCACGGTC-3′ (forward) and 5′-ACGGCTGCTGGACTTCACCA-3′ (reverse); GAPDH, 5′-TGCACCACCAACTGCTTAGC-3′ (forward) and 5′-GGCATGGACTGTGGTCATGAG-3′ (reverse).

### Western blot analysis

Protein lysates were extracted from the cells after the indicated treatments. Western blot experiments were performed as previously described [12].

### Cellular proliferation assay

Cell proliferation was detected by CCK-8 (Dojindo Laboratories, Kumamoto, Japan) assay as previously described [13]. Briefly, the indicated cells were seeded into 96-well plates. CCK-8 assay reagent was added to each well and cultured at 37 °C for 2 h for the indicated times. The optical density (OD) values of the supernatant of each well were then measured in a microplate reader.

### Statistical analysis

All data were analysed by using SPSS software. The values are presented as the mean ± SD. Comparisons between two groups were conducted by t-test. *P* < 0.05 was considered statistically significant.

## Results

### Adipocyte-induced EOC cell proliferation was dependent on SphK1

**Fig. 1:**
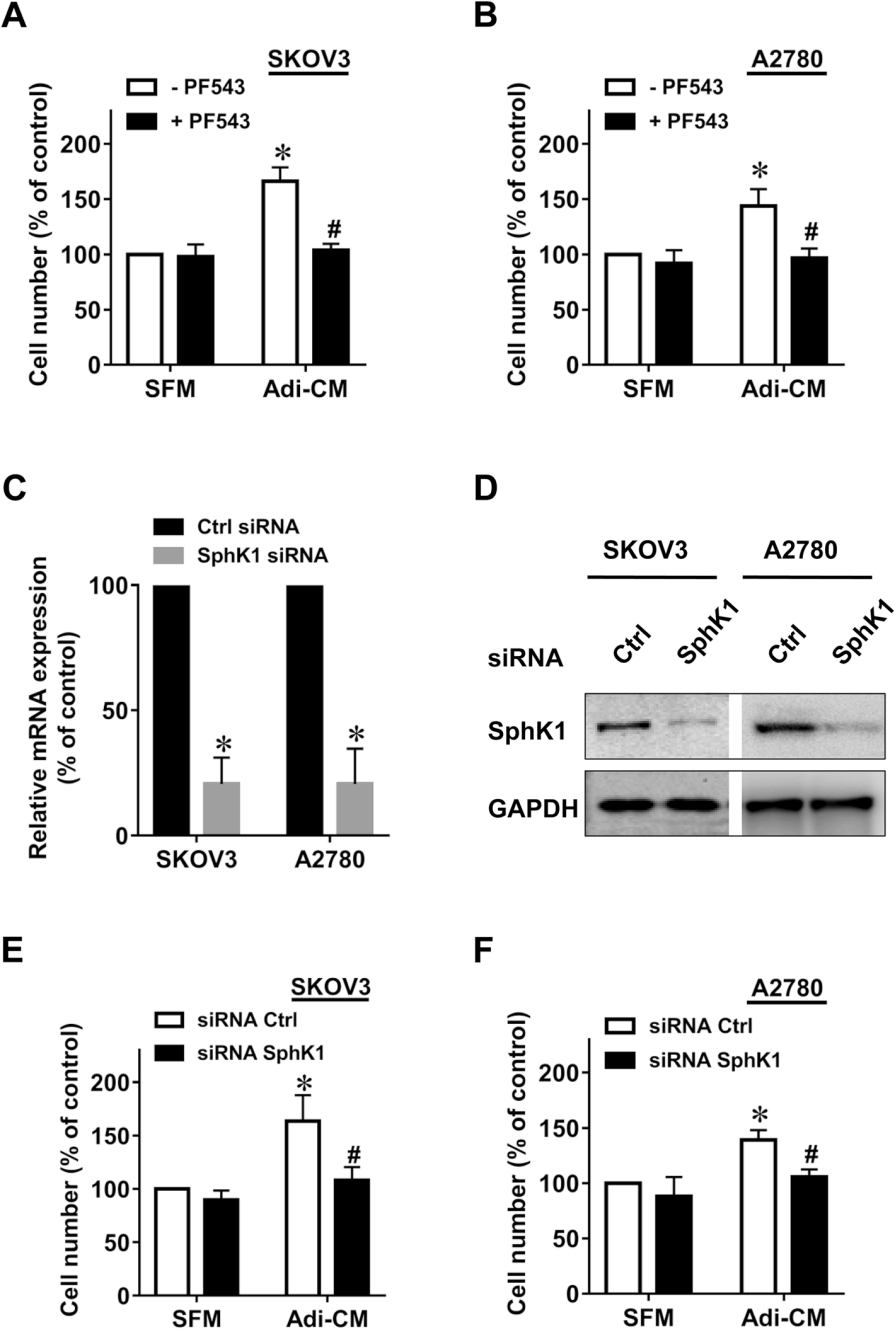
Inhibition of SphK1 suppressed adipocyte-induced EOC cell proliferation. SKOV3 (**a**) and A2780 (**b**) cells were serum starved overnight and cultured with serum-free medium (SFM) or adipocyte-conditioned medium (Adi-CM) in the presence or absence of PF543 (10 μM) for 48 h. Cell proliferation was measured using CCK-8 assay. **c** SphK1 mRNA was measured by quantitative RT-PCR 24 h after siRNA transfection. **d** SphK1 protein levels were determined by western blot analysis 48 h after siRNA transfection. SKOV3 (**e**) and A2780 (**f**) cells were transfected with the indicated siRNAs, followed by culture with SFM or Adi-CM for 48 h. Cell proliferation was measured by CCK-8 assay. The data are presented as the means±SD. *, *P* < 0.05 vs control; #, *P* < 0.05 vs Adi-CM alone

To explore whether adipocytes can induce EOC cell proliferation through the SphK1 pathway, we used PF543 [14], an inhibitor of SphK1. In agreement with previous studies [3], Adi-CM significantly promoted the proliferation rate of EOC cells, as measured by CCK-8 assays (Fig. 1, a-b). Markedly, the SphK1 inhibitor PF543 significantly inhibited Adi-CM-induced proliferation of EOC cell lines. As a control, PF543 alone did not significantly inhibit EOC cell proliferation (Fig. 1, a-b). Moreover, we used siRNA to knockdown SphK1 in ovarian cancer cells. As shown in Fig. 1c-d, both the mRNA and protein levels of SphK1 were significantly reduced after SphK1 siRNA transfection. SphK1 siRNA also significantly inhibited Adi-CM-induced proliferation of EOC cell lines (Fig. 1, e-f). Taken together, these results suggest that SphK1 is critically involved in adipocyte-dependent EOC cell proliferation.

### Adipocytes activated SphK1 in EOC cells

**Fig. 2:**
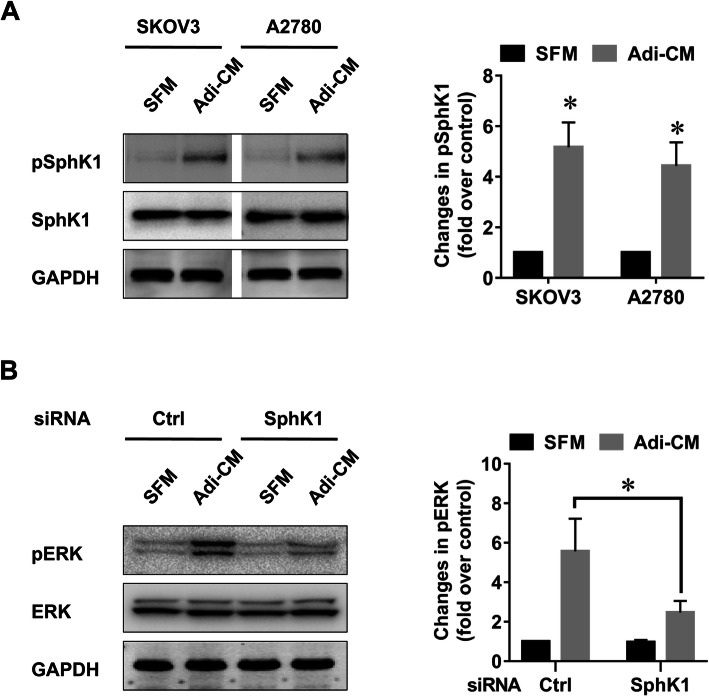
Adipocytes activated SphK1/ERK signal in EOC cells. **a** Serum-starved SKOV3 and A2780 cells were cultured with SFM or Adi-CM for 24 h. SphK1 phosphorylation was determined by western blot analysis. Densitometric analysis of pSphK1 (normalized to total SphK1) is shown on the right. **b** SKOV3 cells were transfected with the indicated siRNAs and cultured with SFM or Adi-CM for 24 h. Levels of total and phosphorylated ERK (pERK) were determined by western blot analysis. The right panel shows densitometric analysis of pERK (normalized to total ERK). The data are presented as the means±SD. *, *P* < 0.05

**Fig. 3:**
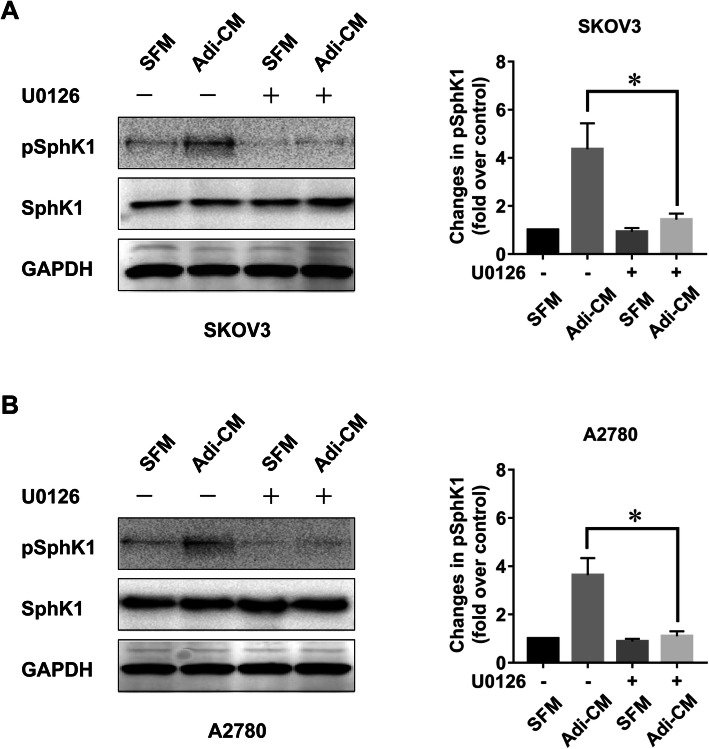
Adipocyte-induced SphK1 activation was ERK dependent. SKOV3 (**a**) and A2780 (**b**) cells were serum starved overnight, pretreated with U0126 (5 μM) for 2 h and then cultured with SFM or Adi-CM for 24 h. Levels of total and phosphorylated SphK1 (pSphK1) were determined by western blot analysis. Right panels show densitometric analysis of pSphK1 (normalized to total SphK1) corresponding to the bands shown in the western blots. The data are presented as the means±SD. *, *P* < 0.05

Given the potential role of SphK1 in adipocyte-mediated EOC cell proliferation, we further explored whether adipocytes can activate SphK1. It has been reported that SphK1 phosphorylation is critical for SphK1 activation [15]. Therefore, we measured the phosphorylation status of SphK1 after Adi-CM treatment. As shown in Fig. 2a, Adi-CM treatment caused an increase in the phosphorylation of SphK1 in EOC cell lines. In addition, Adi-CM activated ERK signalling (Fig. 2b), the major downstream pathway involved in EOC cell proliferation. Moreover, Adi-CM-induced ERK phosphorylation was significantly inhibited by the siRNA-mediated blockade of SphK1 (Fig. 2b), which indicated that SphK1 was involved in adipocyte-induced ERK activation in EOC cells. Previous studies showed that ERK was a critical enzyme causing SphK1 activation [15]. In agreement with this finding, U0126, an inhibitor of the ERK pathway, significantly inhibited Adi-CM-induced SphK1 activation in EOC cells (Fig. 3, a-b). Together, these results indicated that SphK1 can be activated by adipocytes in an ERK-dependent manner in EOC.

### Both S1PR1 and S1PR3 were involved in the growth-promoting action of adipocytes

**Fig. 4:**
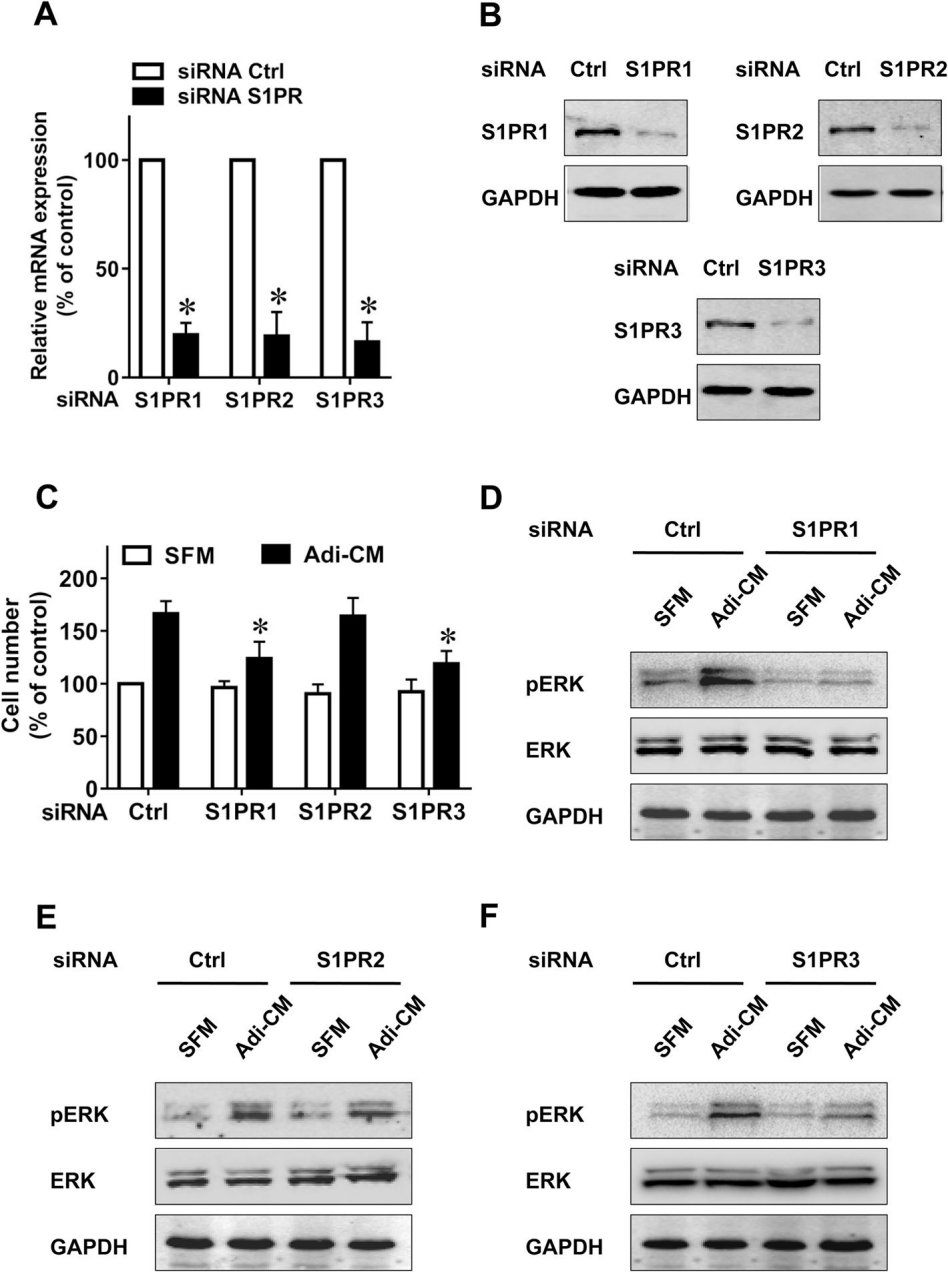
The effect of S1PRs on adipocyte-induced ovarian cancer cell proliferation. **a** SKOV3 cells were transfected with control siRNA or specific siRNA targeting S1PR1–3 as indicated. After 24 h of transfection, the mRNA levels of S1PR1–3 were determined by quantitative RT-PCR. The data are expressed as the change with respect to control siRNA. **b** After 48 h of transfection, the protein levels of S1PR1–3 were determined by western blot analysis and normalized to GAPDH. **c** SiRNA-transfected cells were cultured with SFM or Adi-CM for 48 h. Cell proliferation was measured by CCK-8 assay. **d** to **f** Cells were transfected with the indicated siRNAs and cultured with SFM or Adi-CM for 24 h. Levels of total and phosphorylated ERK (pERK) were then determined by western blot analysis. The data are presented as the means±SD. *, *P* < 0.05

It has been established that the biological consequences of SphK1 activation depend on S1P, which mainly functions by binding S1PRs [16, 17]. We previously reported that S1PR1–3 was overexpressed in EOC tissue [9]. To investigate the role of S1PR1–3 in adipocyte-mediated EOC cell proliferation, we used siRNAs to specifically knockdown S1PR1–3 expression (Fig. 4, a-b). Notably, S1PR1 and S1PR3 downregulation significantly inhibited the EOC cell proliferation induced by Adi-CM (Fig. 4c). In contrast, S1PR2 downregulation did not alter the effects of Adi-CM on EOC cell proliferation (Fig. 4c). Moreover, S1PR1 and S1PR3 knockdown resulted in a significant downregulation of Adi-CM-induced ERK phosphorylation, whereas S1PR2 downregulation had a negligible effect on ERK activation (Fig. 4, d-f). Together, these results suggest the important roles of both S1PR1 and S1PR3 in the growth-promoting effect of adipocytes on EOC cells.

## Discussion

**Fig. 5:**
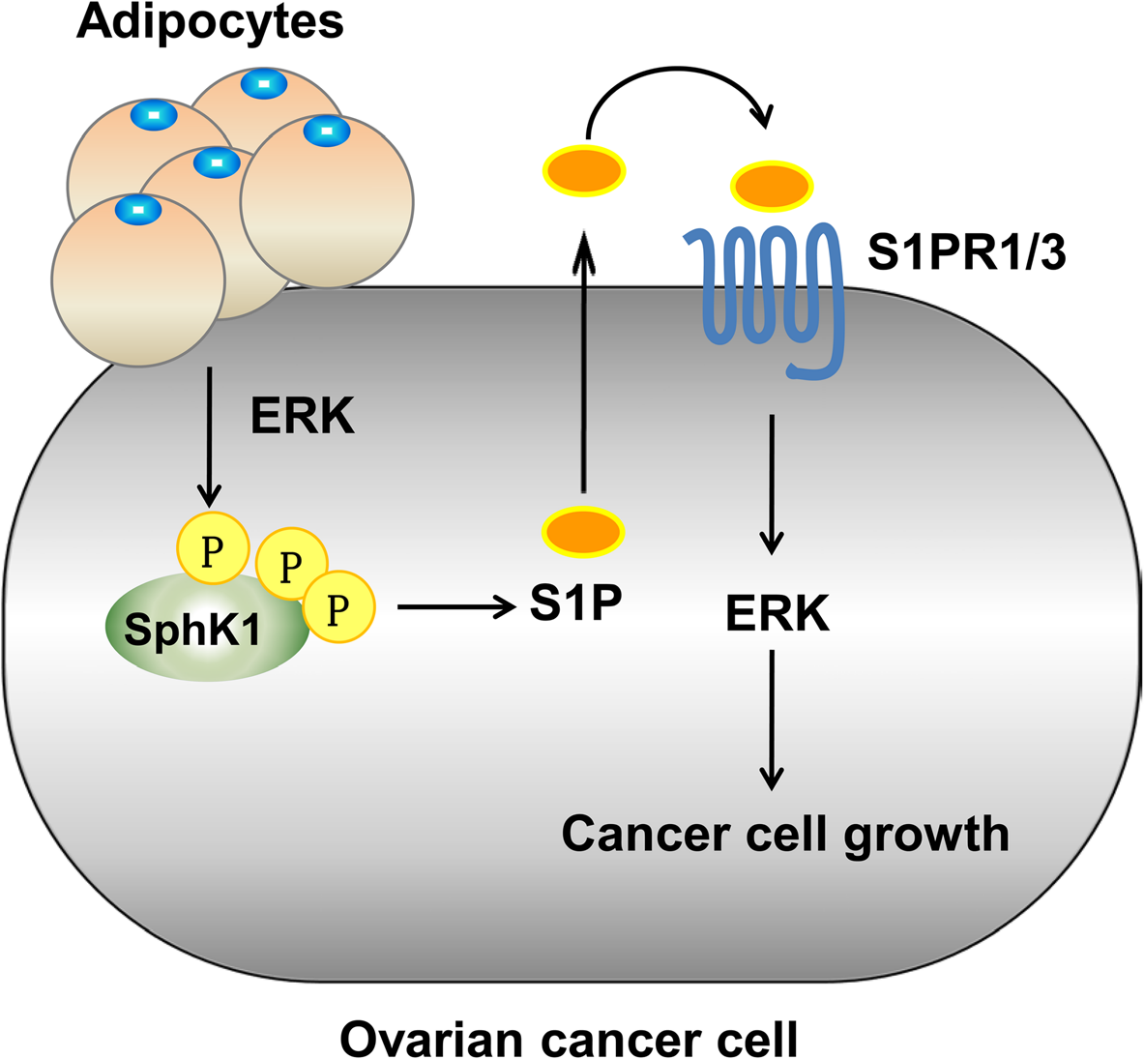
Model of adipocyte-induced EOC cell proliferation through the SphK1-S1PR1/3 signalling pathway. Adipocytes induce ERK-dependent activation of SphK1, release of S1P and the consequent activation of S1PR1/3, leading to ERK phosphorylation and EOC cell proliferation. SphK1 or S1PR1/3 depletion inhibits adipocyte-induced EOC growth

In the current study, we provided mechanistic data delineating a new mechanism that mediated the growth-promoting effects of adipocytes on EOC cells via SphK1 activation. SphK1 blockade suppressed adipocyte-induced EOC growth. Moreover, both S1PR1 and S1PR3, key components in the SphK1 pathway, were critical for adipocyte-induced EOC cell proliferation (summarized in Fig. 5). These results suggest a new target to block adipocyte-induced EOC growth.

Adipocytes are important secretory cells, that release a wide range of adipokines, including hormones, inflammatory cytokines, enzymes and other factors [18, 19]. Many of these adipokines participate in EOC growth promotion by activating a series of signalling pathways in tumour cells [3, 20]. Therefore, we wondered whether there might be one major mediator regulating these adipokines and signalling pathways. We focused on SphK1 because it plays important roles in signal transmission and cancer proliferation [5, 6]. SphK1 overexpression promoted the proliferation of several types of cancer, including ovarian, breast and colon cancer [10, 21, 22]. Moreover, blockade of SphK1 inhibited growth in several tumour types, including breast, colon and thyroid cancer [22–24]. Excitingly, the present study demonstrated, for the first time, the potential role of SphK1 in mediating adipocyte-induced EOC growth. We showed that drug inhibition of SphK1 inhibited adipocyte-dependent EOC cell proliferation. Moreover, adipocyte-induced EOC growth was suppressed by SphK1 specific siRNAs. Furthermore, adipocytes activated SphK1 by inducing SphK1 phosphorylation in EOC cells. Together, these results suggested that SphK1 is not only activated by adipocytes but is also critical for adipocyte-induced cell proliferation in EOC.

The activation of the ERK pathway is recognized as a key cellular phenomenon that initiates and facilitates cell proliferation [25, 26]. ERK activation in EOC cells promoted cell proliferation, and blocking ERK activation inhibited EOC growth [10, 27]. Adi-CM significantly promoted EOC cell proliferation. Therefore, we investigated the role of adipocytes in ERK activation and found that Adi-CM activated ERK through ERK phosphorylation in EOC. Previously, we demonstrated that ERK is a downstream target of SphK1 in EOC [10, 12]. Having shown the ability of adipocytes to activate SphK1, a potential role of SphK1 in adipocyte-induced ERK activation was suggested. Indeed, ERK activation by adipocytes in EOC was significantly reduced by SphK1 blockade. These results indicate a specific role of SphK1 in mediating ERK activation induced by adipocytes. ERK phosphorylation is essential for SphK1 activation [15]. Given that adipocytes activate both ERK and SphK1, we explored the role of ERK in adipocyte-induced SphK1 activation. Indeed, treatment with U0126, a specific inhibitor of the MAPK/ERK pathway, attenuated adipocyte-mediated SphK1 phosphorylation. These results, together with the discovery that SphK1 inhibition results in ERK suppression in EOC cells induced by adipocytes, suggest that ERK might be located both upstream and downstream of SphK1 and may play a dual role in the initiation and amplification of the SphK1 signalling loop.

The biological effects of SphK1 activation chiefly rely on its product, S1P, a bioactive sphingolipid [16, 17]. Previously, we confirmed that SphK1 inhibition or knockdown attenuated S1P production in EOC cells [9]. S1P functions mainly by binding to S1P receptors (S1PRs), including S1PR1 to S1PR5 [16, 17]. We previously found that S1PR1, S1PR2 and S1PR3 were overexpressed in ovarian cancer tissue [9]. Our results showed that S1PR1 or S1PR3 knockdown can partly inhibit adipocyte-induced EOC cell proliferation and ERK activation. However, S1PR2 knockdown had a negligible effect on EOC growth and ERK phosphorylation. Therefore, the effects of S1PR1 and S1PR3, but not S1PR2, were attributed to adipocyte-mediated EOC growth and ERK activation. Although it is well known that the main roles of S1P are mediated through cell surface S1PRs, some important functions of intracellular S1P have also been discovered [28, 29]. Further studies are needed to explore the effects of intracellular S1P on adipocyte-induced EOC growth. Moreover, although S1PR1, S1PR2 and S1PR3 have been reported and proven to be the major receptors that account for the receptor-dependent role of S1P in EOC [30, 31], we cannot exclude the possibility that other members of S1PR, such as S1PR4 or S1PR5, may also be involved in adipocyte-induced cell proliferation. To determine this possibility, further investigation is necessary.

## Conclusions

Our study provides the first evidence that SphK1 plays a crucial role in adipocyte-induced EOC cell proliferation. Adipocytes were capable of activating the SphK1/ERK pathway. Moreover, adipocyte-induced SphK1 activation was ERK dependent. Furthermore, S1PR1 and S1PR3, key components of the SphK1 signalling pathway, participated in adipocyte-mediated growth-promoting action in EOC cells. These data revealed a previously unrecognized role of SphK1 in the adipocyte-induced growth-promoting action in EOC, suggesting a new target for EOC therapy.

## Data Availability

The datasets used and/or analysed during the current study are available from the corresponding author on reasonable request.
